# Trends in the Prevalences of Selected Birth Defects in Korea (2008–2014)

**DOI:** 10.3390/ijerph15050923

**Published:** 2018-05-05

**Authors:** Jung-Keun Ko, Dirga Kumar Lamichhane, Hwan-Cheol Kim, Jong-Han Leem

**Affiliations:** 1 Department of Social and Preventive Medicine, School of Medicine, Inha University, Incheon 22212, Korea; ecokjk@naver.com (J.-K.K.); dirgalamichhane@gmail.com (D.K.L.); cheol17@hanmail.net (H.-C.K.); 2 Department of Occupational and Environmental Medicine, School of Medicine, Inha University, Incheon 22212, Korea

**Keywords:** birth defects, urogenital anomalies, congenital heart defects, prevalence rate ratios, trends

## Abstract

Little information is available on the prevalences of birth defects in Korea. The aims of this study were to estimate recent prevalences of selected birth defects and to analyze the prevalence trends of these defects during the period from 2008 to 2014. Prevalences were calculated for 69 major birth defects using health insurance claim data obtained from the Korea National Health Insurance Service (NHIS). Prevalence rate ratios were calculated using Poisson regression to analyze trends over the 7-year study period. The overall prevalence of a major birth defect was 446.3 per 10,000 births (95% CI: 444.0–448.6); 470.9 per 10,000 births (95% CI: 467.6–474.2) for males and 420.2 per 10,000 births (95% CI: 417–423.4) for females. The prevalence rates of the most common birth defects over the study period were; septal defect (138.2 per 10,000; 95% CI: 136.9–139.5), congenital hip dislocation (652 per 10,000; 95% CI: 64.1–65.9), and ventricular septal defect (62.62 per 10,000; 95% CI: 61.7–63.5). During the study period, a significant increase in the prevalence of a major birth defect was observed with a prevalence rate ratio (PRR) of 1.091. The strongest trend was observed for renal dysplasia, which had a PRR of 1.275 (95% CI: 1.211–1.343), and upward trends were observed for urogenital anomalies, such as, renal agenesis (PRR 1.102, 95% CI: 1.067–1.138), undescended testis (PRR 1.082, 95% CI: 1.072–1.093) and hypospadias (PRR 1.067, 95% CI: 1.044–1.090). This study shows an overall increase in the prevalences of birth defects, including hypospadias and undescended testis, which are known to be associated with endocrine factors. In the future, standardized birth defect registries should be established to enable these trends to be monitored.

## 1. Introduction

Worldwide, about 3–6% of infants are born with a major defect [1], and 11.3% of infant mortalities occurring within four weeks of birth are due to congenital malformations [2]. The European Surveillance of Congenital Anomalies (EUROCAT) reported that the prevalence of major congenital anomalies in 2003–2007 was 239 per 10,000 births, of which 80% were delivered, 17.6% were terminated by induced abortion, 2.5% died after birth, and 2% were stillbirths [3]. During the period 1978–2005, the prevalence of major birth defects was estimated at 3% in Atlanta, GA, USA [4]. In Korea, the prevalence of 27 selected birth defects in 2002 was 57.8 per 10,000 live births and 750.6 per 10,000 stillbirths [5]. The prevalence of 69 major specific birth defects, as monitored by the National Birth Defects Prevention Network (NBDPN), the International Clearinghouse of Birth Defects Surveillance and Research (ICBDSR), and EUROCAT in 2005–2006 were 186.1 and 215.2, respectively [6]. Based on data obtained from 5 hospitals in Seoul and Gyeonggi Province in 2009–2010, the prevalence of the 76 specific birth defects considered by EUROCAT was 348.7 per 10,000 [7].

Trends of congenital anomalies in Europe during 1999–2008 showed abdominal wall, gastroschisis, hypospadias, trisomy 18 and renal dysplasia significantly increased, and that neural tube defect (NTD), anophthalmos/microphthalmos, severe congenital heart defects (CHDs) and limb reduction significantly decreased, and suggested decreases in neural tube defects were due to improved periconceptional folic acid supplementation [8]. Another study reported that the prevalence of NTD was lower in countries providing folic acid [9]. In Glasgow (UK) most anomalies declined from 1980 to 1997, whereas chromosomal anomalies showed an increase trend [10]. Cleft lip with or without cleft palate was found to show no significant change in US, but to follow a declining trend globally [11]. The prevalence of CHD has increased considerably over time worldwide, and recently it was reported Asia has the highest prevalence of CHDs [12]. In Korea, cryptorchidism increased from 5.01 to 17.43 per 10,000 births and hypospadia also increased from 1.40 to 3.28 per 10,000 births between 2000 and 2005 [13]. In addition, a Korean study based on medical insurance claims data for 2009–2010 showed a 14-fold increase in hypospadias and epispadias, a 12-fold increase in atrial septal defects, a 11-fold increase in undescended testis as compared with 1993–1994 [14].

The causes of 65–75% of congenital malformations are unknown, of the remainder 15–25% are caused by genetic factors and 10% are attributed to environmental factors [15]. Potential risk factors include chemical exposure, nutritional imbalance, ionizing radiation, and infection [16]. In particular, environmental epidemiological studies have focused on relations between types of environmental pollution and congenital anomalies. Several authors have suggested associations between air pollution and selected birth defects, including CHD, spina bifida and cleft palate [17,18,19,20]. Furthermore, CHDs have been reported to cluster in industrial areas [21], and increased risks of spina bifida, cleft palate, and obstructive heart defects have been reported in areas where maternal residence is near sources of chlorinated solvent emissions [22]. Some studies have reported relations between maternal exposure to endocrine disruptors and genital-urinary defects, such as, cryptorchidism, hypospadias, and urinary tract defects [23,24,25,26,27]. Although few studies have addressed relations between environmental pollution and congenital anomalies, interest in associations between CHD and genital-urinary anomalies and air pollution and exposure to endocrine disruptors are increasing.

No recent data is available on the prevalences of birth defects in Korea. The detection of trends can indicate the emergence of new teratogens and are valuable for the epidemiological surveillance of birth defects and for identifying and issuing warnings about environmental risk factors. The aims of this study were to estimate the recent prevalence of selected birth defects and to analyze the prevalence trends of selected birth defects during the period 2008–2014.

## 2. Methods

We used health insurance claim data from the Korea National Health Insurance Service (NHIS). On the NHISS database, the subjects were congenital anomalies are coded according to the International Classification of Diseases, 10th Revision (ICD-10) within the first year after birth. We investigated 69 major birth defects, which were also monitored by NBDPN, ICBDSR, and EUROCAT. The study period was 2008–2014.

The prevalences of birth defects were calculated by dividing the number of registered birth defects by the total number of births and expressing this as the number of cases per 10,000 live births. The Poisson distribution was used to calculate 95% confidence intervals for prevalences, and prevalence rate ratios were calculated using Poisson regression to analyze trends over the study period. R version 3.40 (The Comprehensive R Archive Network: http://cran.r-project.org) was used for the data analysis.

## 3. Results

### 3.1. Prevalences of Birth Defects During the Study Period

Table 1 shows the prevalences of specific types of major birth defects in Korea over the study period (2008–2014). During this period, the number of live births was 3,208,617, of which 1,650,689 (51.4%) were males and 1,557,928 (48.6%) were females. The prevalence of a major birth defect was 446.3 per 10,000 births (95% CI: 444.0–448.6); 470.9 per 10,000 births (95% CI: 467.6–474.2) for males and 420.0 per 10,000 births (95% CI: 417–423.4) for females. The five most common birth defects were atrial septal defect (138.2 per 10,000; 95% CI: 136.9–139.5), congenital hip dislocation (652 per 10,000; 95% CI: 64.1–65.9), ventricular septal defect (62.62 per 10,000; 95% CI: 61.7–63.5), undescended testis (60.82 per 10,000; 95% CI: 59.6–62), and obstructive genitourinary defect (41.72 per 10,000; 95% CI: 41–42.4).

### 3.2. Congenital Anomalies of the Central Nervous System

**Figure 1:**
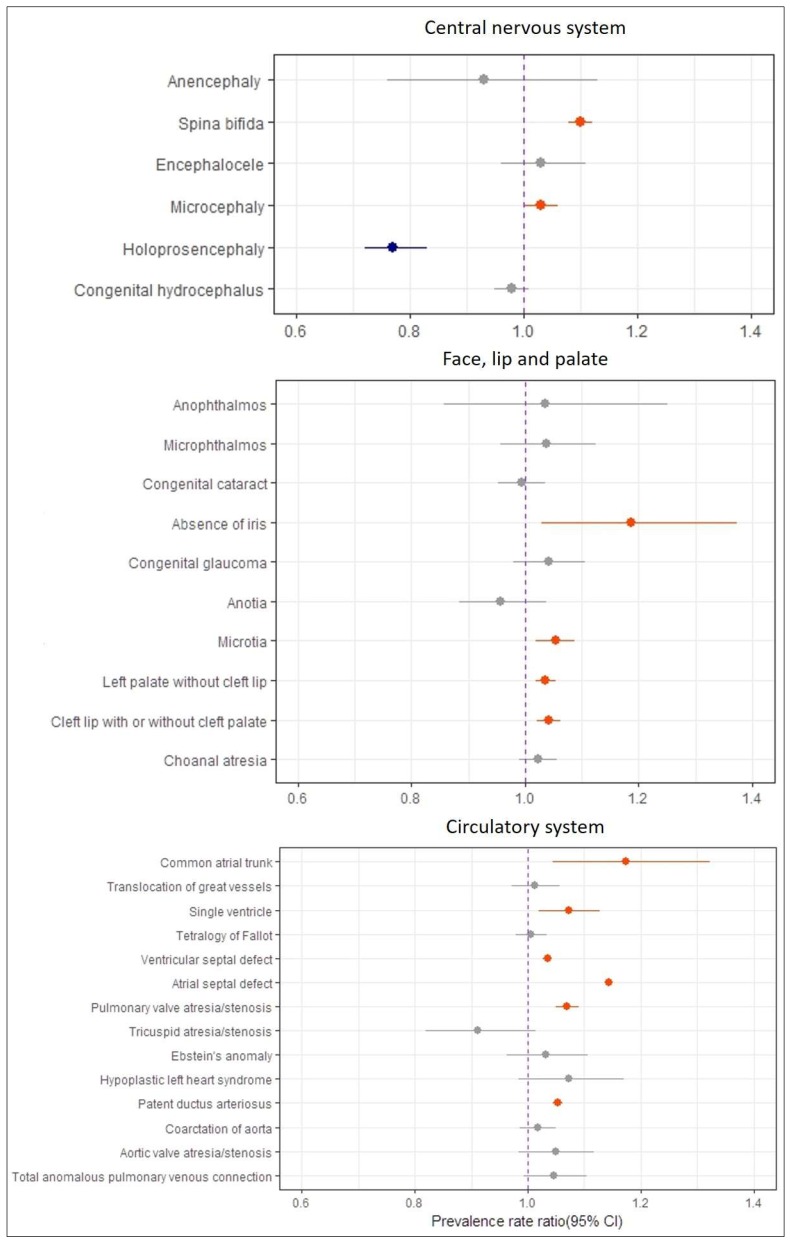
Prevalence rate ratio of congenital anomalies of central nervous system, eye face lip and palate, and circulatory system with 95% CI in Korea, 2008–2014.

The most common birth defects were spina bifida (8.1 per 10,000; 95% CI: 7.6–8.5), microcephaly (3.2 per 10,000; 95% CI: 3.0–3.4), and congenital hydrocephalus (2.9 per 10,000; 95% CI: 2.7–3.0) (Table 1). The prevalence trends of congenital anomalies of the central nervous system are provided in Figure 1. The prevalences of spina bifida (PRR 1.10, 1.08–1.12) and microcephaly (PRR 1.03, 1.00–1.06) increased significantly over the study period, whereas the prevalence of holoprosencephaly decreased significantly. Other birth defects revealed no significant trends.

### 3.3. Congenital Anomalies of Eye, Ear, Face and Neck

The most common birth defects were microtia (2.9 per 10,000; 95% CI: 2.7–3.1), congenital cataract (1.8 per 10,000; 95% CI: 0.7–2.0), and congenital glaucoma (0.83 per 10,000; 95% CI: 0.74–0.94). The prevalence of anophthalmos was 0.09 per 10,000 births (95% CI: 0.06–0.13). In addition, 150 cases of microphthalmos (0.47 per 10,000; 95% CI: 0.40–0.55), 51 cases of absence of iris (0.16 per 10,000; 95% CI: 0.12–0.21), and 150 cases of anotia (0.47 per 10,000; 95% CI: 0.40–0.55) were reported (Table 1). While the prevalences of absence of iris (PRR 1.186, 1.06–1.373) and microtia (PRR 1.053, 1.019–1.088) increased significantly, those of other birth defects remained stable through the study period (Figure 1).

### 3.4. Congenital Anomalies of Lip and Palate

Prevalences of cleft palate without cleft lip, cleft lip with or without cleft palate, and choanal atresia were 9.3 per 10,000 births (95% CI: 9.0–9.7), 7.2 per 10,000 births (95% CI: 6.9–7.5), and 0.45 per 10,000 births (95% CI: 0.38–0.53), respectively (Table 1). During the study period, increasing trends of 3.6% (95% CI: 1.8–5.5) and 4.1% (95% CI: 2.0–6.3) per annum were observed for cleft palate without cleft lip and cleft lip with or without cleft palate (Figure 1).

### 3.5. Congenital Anomalies of the Circulatory System

The three most common birth circulatory system defects were atrial septal defect (138.2 per 10,000; 95% CI: 136.9–139.5), ventricular septal defect (62.6 per 10,000; 95% CI: 61.7–63.5), and patent ductus arteriosus (41.2 per 10,000; 95% CI: 40.5–41.9) (Table 1). The prevalence of congenital heart defects in females was greater than that in males. Prevalence trend analyses showed significant increasing trends for common atrial trunk (PRR 1.173, 95% CI: 1.045–1.322), single ventricle (PRR 1.072, 95% CI: 1.019–1.128), ventricular septal defect (PRR 1.035, 95% CI: 1.027–1.042), atrial septal defect (PRR 1.143, 95% CI: 1.138–1.149), pulmonary valve atresia/stenosis (PRR 1.07, 95% CI: 1.05–1.09), and patent ductus arteriosus (PRR 1.053, 95% CI: 1.044–1.062) over the study period (Figure 1).

### 3.6. Congenital Anomalies of the Digestive System

**Figure 2:**
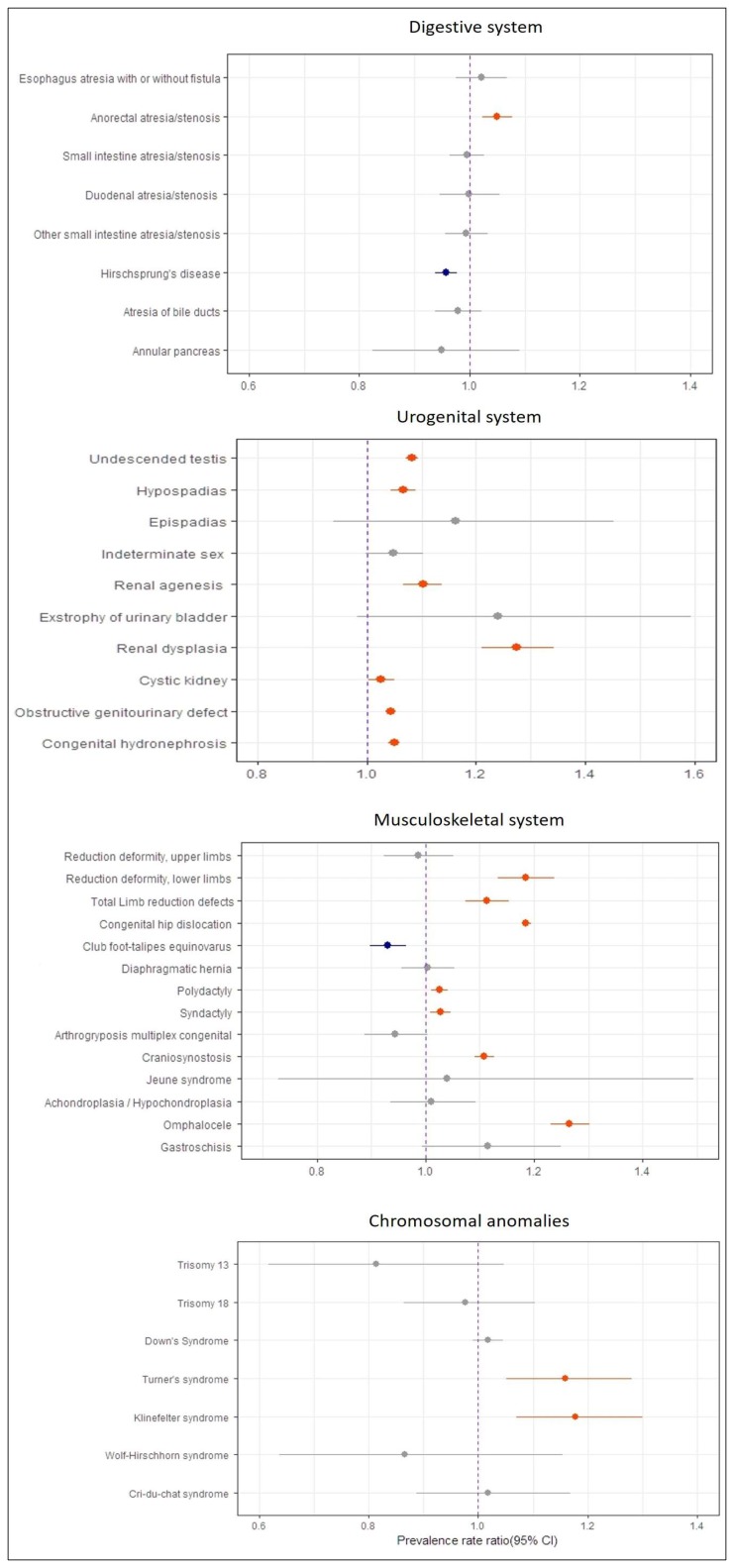
Prevalence rate ratio of congenital anomalies of digestive, chromosomal, musculoskeletal, and urogenital system with 95% CI in Korea, 2008–2014.

Hirschsprung’s disease (7.4 per 10,000; 95% CI: 7.2–7.8), anorectal atresia/stenosis (4.5 per 10,000; 95% CI: 4.3–4.8), and small intestine atresia/stenosis (3 per 10,000; 95% CI: 2.8–3.2) were most prevalent anomalies of the digestive system (Table 1). The prevalence of anorectal atresia/stenosis increased slightly during the study period (PRR 1.05, 1.023–1.078) (Figure 2). However, the prevalence of Hirschsprung’s disease showed a mild decrease (PRR 0.957, 0.938–0.977). Other anomalies were fairly stable over the study period.

### 3.7. Congenital Anomalies of the Urogenital System

The most common anomalies of the urogenital system were undescended testis (60.8 per 10,000 boys; 95% CI: 59.6–62), obstructive genitourinary defect (41.7 per 10,000; 95% CI: 41.0–42.4), congenital hydronephrosis (33.4 per 10,000; 95% CI: 32.8–34), and hypospadias (12.7 per 10,000 boys; 95% CI: 12.2–13.3). The prevalences of obstructive genitourinary defect and congenital hydronephrosis were greater among males than females. In addition, 392 cases of indeterminate sex (1.2 per 10,000; 95% CI: 1.1–1.3), 945 cases of renal agenesis (2.9 per 10,000; 95% CI: 2.8–3.1), and 418 cases of renal dysplasia (1.3 per 10,000; 95% CI: 1.2–1.4) were reported. Furthermore, most urogenital anomalies showed a significant increase of the study period (Figure 2); undescended testis (PRR 1.082, 95% CI: 1.072–1.093), hypospadias (PRR 1.067, 95% CI: 1.044–1.090), renal agenesis (PRR 1.102, 95% CI: 1.067–1.138), cystic kidney (PRR 1.026, 95% CI: 1.002–1.051), obstructive genitourinary defect (PRR 1.044, 95% CI: 1.035–1.053), and congenital hydronephrosis (PRR 1.05, 95% CI: 1.04–1.06). Renal dysplasia showed the greatest increasing trend (PRR 1.275, 95% CI: 1.211–1.343) among the 69 major birth defects over the study period (Table 2).

### 3.8. Congenital Anomalies of the Musculoskeletal System

In the present study, the three most common anomalies of the musculoskeletal system were congenital hip dislocation (65.0 per 10,000; 95% CI: 64.1–65.9), polydactyly (14.1 per 10,000; 95% CI: 13.7–14.5) and craniosynostosis (12.1 per 10,000; 95% CI: 11.7–12.5) (Table 1). Statistically significant upward trends were observed for reduction deformity (lower limbs), total limb reduction, congenital hip dislocation, polydactyly, syndactyly, craniosynostosis, and omphalocele during the study period (Figure 2). However, the prevalence of club foot-talipes equinovarus showed downward trends. Other anomalies of the musculoskeletal system, notably achondroplasia, omphalocele, Jeune syndrome, reduction deformity (upper limbs), diaphragmatic hernia, and gastroschisis were stable through the study period.

### 3.9. Chromosomal Anomalies

Down syndrome (4.1 per 10,000; 95% CI: 3.8–4.3) was the most prevalent chromosomal anomaly. During the study period, the prevalence of Turner’s syndrome was 0.33 per 10,000 births (95% CI: 0.27–0.40), Klinefelter’s syndrome was 0.34 per 10,000 births (95% CI: 0.28–0.41), Trisomy 18 was 0.21 per 10,000 births (95% CI: 0.16–0.27), and Cri-du-chat syndrome was 0.16 per 10,000 births (95% CI: 0.12–0.21) (Table 1). In addition, increasing trends of 15.9% (95% CI: 5.2–28%) and 17.8% (95% CI: 7–30%) per year were observed for Turner’s syndrome and Klinefelter’s syndrome, respectively. Other chromosomal anomalies showed no significant trend changes over the study period (Figure 2).

### 3.10. Ranking of Increasing Trends in Birth Defect Subtypes

Table 2 shows the increasing trends exhibited by the ten major birth defects over the study period. Renal dysplasia showed the greatest increase (PRR = 1.275, 95% CI: 1.211–1.343), followed by omphalocele (PRR = 1.265, 95% CI: 1.230–1.302), congenital hip dislocation (PRR = 1.185, 95% CI: 1.177–1.194), reduction deformity of lower limbs (PRR = 1.184, 95% CI: 1.133–1.237), and atrial septal defect (PRR = 1.143, 95% CI: 1.138–1.149).

## 4. Discussion

In the current study, a significant increase in the prevalence of 69 major birth defects with a prevalence rate ratio (PRR) of 1.091 was observed during the study period (2008–2014) in Korea, which means that the overall prevalence of birth defects increased by 9.1% per year on average. We identified apparent upward trends in the prevalences of urogenital anomalies, particularly, renal dysplasia, renal agenesis, undescended testis, and hypospadias. In Korea, previous studies have reported increases in the prevalence of some urogenital anomalies [13,14]. Kim et al. indicated that the incidence of cryptorchidism and hypospadias increased in Korea from 2000 to 2005 (5.01 to 17.43 per 10,000 and 1.40 to 3.28 per 10,000 births, respectively). Lamichhane et al. also reported that the prevalence of some urogenital birth defects increased from 14.1 to 6.2 times between 1993–1994 and 2009–2010. Hence, our study suggests that urogenital birth defects persist at high levels in Korea.

Trends in the prevalences of urogenital anomalies vary among countries. In the EU (1999–2008), renal dysplasia increased (3.8% increase per 2 years), but congenital hydronephrosis decreased (6.8% decrease per 2 years) [8]. While trends in the incidences of congenital anomalies of the kidney and urinary tract (CAKUT) remained stable in Taiwan (2004–2011) [28], a decreasing trend (−31%) was reported in Glasgow (UK) (1980–1997) [10]. With regard to hypospadias and cryptorchidism, no change in the prevalence of hypospadias was observed in Nova Scotia (Canada) [29], but an increasing trend was reported in the EU (1999–2008) [8]. Furthermore, the prevalence cryptorchidism showed a slightly decreasing trend in Nova Scotia (1988–2013) [29], but increasing trends in the UK and Denmark (1950s–2000s) [30].

Urinary malformations are caused by complex interactions between genetic and non-genetic factors, some of which have been identified [31]. Some studies have suggested that maternal diabetes, kidney disease, smoking during pregnancy, and drinking influence urinary anomalies [32,33], whereas others have suggested environmental pollution is an important factor. Studies on environmental pollution indicate the risks of renal dysplasia and obstructive genitourinary defect are higher in regions with a dioxin-emitting incinerator [26,27], and suggest an association between the chlorination disinfection byproduct in drinking water and urinary anomalies [34]. Abbott et al. concluded that TCDD (2,3,7,8-tetracholrodibenzo-p-dioxin) induces hydronephrosis in mice [35], and others have suggested an association between congenital renal defects and low birth weight (LBW) in animal models and human [36,37]. Chevalier et al. suggested genetic defects in hormone synthesis and receptors related to testicular descent, endocrine disrupting chemicals (EDCs), smoking, and drinking were causes of undescended testis [37]. Koskenniemi et al. suggested that exposure to dioxin increased the risk of undescended testis [23], whereas others suggested that exposure to EDCs and organic solvents increased the risk of hypospadias [24,25].

The present study shows that the most common birth defects of the circulatory system were atrial septal defect (138.2 per 10,000; 95% CI: 136.9–139.5), congenital hip dislocation (65.2 per 10,000; 95% CI: 64.1–65.9), and ventricular septal defect (62.62 per 10,000; 95% CI: 61.7–63.5). This finding is consistent with those of previous studies that showed atrial septal defect and ventricular septal defect are the most common defects in Korea [14,38]. Congenital heart defect (CHD) had the highest prevalence in the present study, and reportedly is the most common anomalies in other countries [12,39]. EUROCAT (2011–2015) reported that among congenital anomalies ventricular septal defect had the highest prevalence, followed by hypospadias and atrial septal defect [40]. In the US, the common defects were hypospadias, atrial septal defect, and ventricular septal defect during 2008–2012 [41]. Although these rates differ from those obtained in the current study, CHD proportions were similar.

Furthermore, the current study shows the prevalence of some CHD subtypes increased at 3.4–14.3% per year during the study period (2008–2014). A meta-analysis indicated that the increase in total CHD worldwide showed a steep increase from the late 1970s until 1995, and stabilized from 1995 [12]. The authors suggested that these increases may have been due to improvements in diagnostic techniques, such as, ultrasound, and screening methods. Cavadino et al. reported decreasing trends in the prevalences of atrial septal defect and pulmonary valve stenosis, but no significant trend for ventricular septal defect in EUROCAT (2003–2012) [42]. The authors suggested that trend differences might be due to different case definitions used in the EUROCAT study. In a Norwegian study (1994–2009), CHDs showed a significant increase until around 2005 and a subsequent decrease. The authors suggested that increased consumption of folic acid may have explained this decreasing trend [43]. In addition, the following have been reported to be risk factors of CHDs; genetic abnormalities, maternal illnesses, and maternal factors, such as, maternal diabetes, rubella, febrile illnesses and obesity, drug exposure, smoking, drinking alcohol, environmental exposure, and maternal sociodemographic characteristics [44]. Recently, the number of environmental epidemiological papers published on CHDs has increased. Some studies have shown relations between CHDs and environmental pollution, such as, air pollution and chemicals [18,19,22]. Tanner et al. reported a positive relationship between exposure to PM_2.5_ and increased CHD prevalence in Florida in a retrospective cohort study [17], and Zhang et al. suggested the risk of ventricular septal defect increased 1.11–1.17 per 10 μm/m^3^ change in PM_2.5_ during the 7th to 10th weeks of pregnancy [45]. Others have suggested that phthalate exposure is associated with the risk of CHDs, such as, ventricular septal defect and atrial septal defect [46,47], and Gilboa et al. found that occupational exposure to chlorinated organic solvents was related to ventricular septal defect [48].

In the present study, the overall prevalence of omphalocele was 4.2 per 10,000 births per annum, which is higher than that reported in the US (1995–2005) [49], and was the second highest increasing trend (26.6% increase per annum) observed in the present study. Congenital hip dislocation was found to be a common defect (65 per 10,000 births), and also showed a considerable increasing trend (18.5% increase per year). Prevalences of congenital hip dislocation vary by country from 1.5 to 4.9% [50]. Its causes have been suggested to be breech presentation, oligohydramnios, skeletal malformations due to teratogenic agents, and neuromuscular disease [51].

The strength of the present study is that it had substantial statistical power because it was conducted using nationally representative data from the NHIS. The national health insurance system in Korea covers almost all of the population, and thus, the use of NHIS data minimizes selection-associated risks and related potential bias. In addition, the dataset analyzed provided sufficient power to analyze trends. However, there were several limitations to this study. First, the prevalences were likely to be underestimated due to using the data that did not include stillbirth, miscarriage, and abortion. Second, the present study is subject to limitations associated with the use of insurance claims data, particularly with respect to coding, diagnostic criteria and the timings for follow-up of patients, which differ between hospitals. Furthermore, it was difficult to validate accuracies of diagnoses using claim data due to a lack of detailed records. Finally, it was difficult to adjust for maternal characteristics (e.g., maternal age) affecting the trends in the prevalence because the NHIS data could not be merged with the birth and maternal data due to the privacy protection.

## 5. Conclusions

The current study shows the prevalence of major birth defects increased significantly in Korea from 2008 to 2014. In particular, apparent upward trends in the prevalences of hormone-mediated urogenital anomalies, such as, undescended testis and hypospadias, may suggest the involvement of environmental factors. Furthermore, congenital heart defects also showed increasing trends over the study period, possibly due to the development of diagnostic techniques and more frequent prenatal diagnosis. However, increasing trends were not consistent across all categories of birth defects, as significant decreases were observed in the prevalences of holoprosencephaly, Hirschsprung’s disease and club foot-talipes equinovarus. In future, it is needed to establish a nationwide surveillance system for birth defects to evaluate the risk factors for congenital anomalies and prevention efforts.

## Figures and Tables

**Table 1:** Prevalences of specific types of major birth defects in Korea, 2008–2014.

Birth Defects(ICD-10)	Total		Male		Female	
	Number of Cases	Prevalence Per 10,000 (95%CI)	Number of Cases	Prevalence Per 10,000 (95%CI)	Number of Cases	Prevalence Per 10,000 (95%CI)
Birth	3,208,617	-	1,650,689	-	1,557,928	-
Overall major birth defect	143,196	446.3 (444–448.6)	77,733	470.9 (467.6–474.2)	65463	420.2 (417–423.4)
Central nervous system						
Anencephaly (Q00.0–00.2)	25	0.08 (0.05–0.12)	15	0.09 (0.05–0.15)	10	0.06 (0.03–0.12)
Spina bifida (Q05.0–05.9)	2759	8.6 (8.3–8.9)	1330	8.1 (7.6–8.5)	1429	9.2 (8.7–9.7)
Encephalocele (Q01.0–01.9)	182	0.57 (0.49–0.66)	91	0.55 (0.44–0.68)	91	0.58 (0.47–0.72)
Microcephaly (Q02)	1013	3.2 (3–3.4)	467	2.8 (2.6–3.1)	546	3.5 (3.2–3.8)
Holoprosencephaly (Q04.0–04.2)	251	0.78 (0.69–0.89)	151	0.91 (0.77–1.07)	100	0.64 (0.52–0.78)
Congenital hydrocephalus (Q03.0–03.9)	917	2.9 (2.7–3)	561	3.4 (3.1–3.7)	356	2.3 (2.1–2.5)
Eye, ear, face and neck						
Anophthalmos (Q11.0–11.1)	28	0.09 (0.06–0.13)	15	0.09 (0.05–0.15)	13	0.08 (0.04–0.14)
Microphthalmos (Q11.2)	150	0.47 (0.4–0.55)	86	0.52 (0.42–0.64)	64	0.41 (0.32–0.52)
Congenital cataract (Q12.0)	581	1.8 (1.7–2)	302	1.8 (1.6–2)	279	1.8 (1.6–2)
Absence of iris (Q13.1)	51	0.16 (0.12–0.21)	28	0.17 (0.11–0.25)	23	0.15 (0.09–0.22)
Congenital glaucoma (Q15.0)	267	0.83 (0.74–0.94)	159	0.96 (0.82–1.13)	108	0.69 (0.57–0.84)
Anotia (Q16.0)	150	0.47 (0.4–0.55)	93	0.56 (0.45–0.69)	57	0.37 (0.28–0.47)
Microtia (Q17.2)	929	2.9 (2.7–3.1)	580	3.5 (3.2–3.8)	349	2.2 (2–2.5)
Lip, plate						
Cleft palate without cleft lip (Q35.1–35.9)	3000	9.3 (9–9.7)	1341	8.1 (7.7–8.6)	1659	10.6 (10.1–11.2)
Cleft lip with or without cleft palate (Q36.0–37.9)	2309	7.2 (6.9–7.5)	1395	8.5 (8–8.9)	914	5.9 (5.5–6.3)
Choanal atresia (Q30.0)	144	0.45 (0.38–0.53)	77	0.47 (0.37–0.58)	67	0.43 (0.33–0.55)
Circulatory system						
Common atrial trunk (Q20.0)	75	0.23 (0.18–0.29)	34	0.21 (0.14–0.29)	41	0.26 (0.19–0.36)
Translocation of great vessels (Q20.3)	574	1.8 (1.6–1.9)	415	2.5 (2.3–2.8)	159	1.02 (0.87–1.19)
Single ventricle (Q20.4)	386	1.2 (1.1–1.3)	214	1.3 (1.1–1.5)	172	1.1 (0.9–1.3)
Tetralogy of Fallot (Q21.3)	1365	4.3 (4–4.5)	777	4.7 (4.4–5.1)	588	3.8 (3.5–4.1)
Ventricular septal defect (Q21.0)	20,082	62.6 (61.7–63.5)	9466	57.3 (56.2–58.5)	10,616	68.1 (66.9–69.5)
Atrial septal defect (Q21.1)	44,335	138.2 (136.9–139.5)	21,544	130.5 (128.8–132.3)	22,791	146.3 (144.4–148.2)
Pulmonary valve atresia/stenosis (Q22.0, Q22.1)	2846	8.9 (8.5–9.2)	1312	7.9 (7.5–8.4)	1534	9.8 (9.4–10.4)
Tricuspid atresia/stenosis (Q22.4)	88	0.27 (0.22–0.34)	51	0.31 (0.23–0.41)	37	0.24 (0.17–0.33)
Ebstein’s anomaly (Q22.5)	205	0.64 (0.55–0.73)	109	0.66 (0.54–0.8)	96	0.62 (0.5–0.75)
Hypoplastic left heart syndrome (Q23.4)	130	0.41 (0.34–0.48)	77	0.47 (0.37–0.58)	53	0.34 (0.25–0.44)
Patent ductus arteriosus (Q25.0)	13,209	41.2 (40.5–41.9)	6364	38.6 (37.6–39.5)	6845	43.9 (42.9–45)
Coarctation of aorta (Q25.1)	979	3.1 (2.9–3.2)	571	3.5 (3.2–3.8)	408	2.6 (2.4–2.9)
Aortic valve atresia/stenosis (Q23.0)	249	0.78 (0.68–0.88)	168	1 (0.9–1.2)	81	0.52 (0.41–0.65)
Total anomalous pulmonary venous connection (Q26.2)	349	1.1 (1–1.2)	209	1.3 (1.1–1.4)	140	0.9 (0.76–1.06)
Digestive system						
Esophagus atresia with or without fistula (Q39.0, Q39.1)	484	1.5 (1.4–1.6)	286	1.7 (1.5–1.9)	198	1.3 (1.1–1.5)
Anorectal atresia/stenosis (Q42.0–42.3)	1456	4.5 (4.3–4.8)	819	5 (4.6–5.3)	637	4.1 (3.8–4.4)
Small intestine atresia/stenosis (Q41.0–41.9)	968	3 (2.8–3.2)	498	3 (2.8–3.3)	470	3 (2.8–3.3)
Duodenal atresia/stenosis (Q41.0)	325	1 (0.9–1.1)	148	0.9 (0.76–1.05)	177	1.1 (1–1.3)
Other small intestine atresia/stenosis (Q41.1–41.9)	663	2.1 (1.9–2.2)	361	2.2 (2–2.4)	302	1.9 (1.7–2.2)
Hirschsprung’s disease (Q43.1)	2390	7.4 (7.2–7.8)	1338	8.1 (7.7–8.6)	1052	6.8 (6.4–7.2)
Atresia of bile ducts (Q44.2)	531	1.7 (1.5–1.8)	248	1.5 (1.3–1.7)	283	1.8 (1.6–2)
Annular pancreas (Q45.1)	51	0.16 (0.12–0.21)	16	0.1 (0.06–0.16)	35	0.22 (0.16–0.31)
Genital organs						
Undescended testis (Q53.0–53.9)	10,033	31.3 (30.7–31.9)	10,031	60.8 (59.6–62)	2	0.01 (0–0.05)
Hypospadias (Q54.0–54.9)	2104	6.6 (6.3–6.8)	2102	12.7 (12.2–13.3)	2	0.01 (0–0.05)
Epispadias (Q64.0)	23	0.07 (0.05–0.11)	22	0.13 (0.08–0.2)	1	0.01 (0–0.04)
Indeterminate sex (Q56.0–56.4)	392	1.2 (1.1–1.3)	239	1.4 (1.3–1.6)	153	0.98 (0.83–1.15)
Urinary system						
Renal agenesis (Q60.0–60.6)	945	2.9 (2.8–3.1)	419	2.5 (2.3–2.8)	526	3.4 (3.1–3.7)
Exstrophy of urinary bladder (Q64.1)	19	0.06 (0.04–0.09)	8	0.05 (0.02–0.1)	11	0.07 (0.04–0.13)
Renal dysplasia (Q61.4)	418	1.3 (1.2–1.4)	174	1.1 (0.9–1.2)	244	1.6 (1.4–1.8)
Cystic kidney (Q61.0–61.9)	1751	5.5 (5.2–5.7)	830	5 (4.7–5.4)	921	5.9 (5.5–6.3)
Obstructive genitourinary defect (Q62.0–62.8, Q64.3)	13,377	41.7 (41–42.4)	9906	60 (58.8–61.2)	3471	22.3 (21.5–23)
Congenital hydronephrosis (Q62.0)	10,719	33.4 (32.8–34)	8171	49.5 (48.4–50.6)	2548	16.4 (15.7–17)
Musculoskeletal system						
Reduction deformity, upper limbs (Q71.0–71.9)	234	0.73 (0.64–0.83)	159	0.96 (0.82–1.13)	75	0.48 (0.38–0.6)
Reduction deformity, lower limbs (Q72.0–72.9)	543	1.7 (1.6–1.8)	237	1.4 (1.3–1.6)	306	2 (1.8–2.2)
Total Limb reduction defects/include unspecified (Q71.0–71.9, Q72.0–72.9, Q73.0–73.8)	793	2.5 (2.3–2.6)	409	2.5 (2.2–2.7)	384	2.5 (2.2–2.7)
Congenital hip dislocation (Q65.0–65.9)	20,858	65 (64.1–65.9)	7410	44.9 (43.9–45.9)	13,448	86.3 (84.9–87.8)
Club foot—talipes equinovarus (Q66.0)	744	2.3 (2.2–2.5)	388	2.4 (2.1–2.6)	356	2.3 (2.1–2.5)
Diaphragmatic hernia (Q79.0)	408	1.3 (1.2–1.4)	236	1.4 (1.3–1.6)	172	1.1 (0.9–1.3)
Polydactyly (Q69.0–69.9)	4534	14.1 (13.7–14.5)	2594	15.7 (15.1–16.3)	1940	12.5 (11.9–13)
Syndactyly (Q70.0–70.9)	2756	8.6 (8.3–8.9)	1590	9.6 (9.2–10.1)	1166	7.5 (7.1–7.9)
Arthrogryposis multiplex congenital (Q74.3)	265	0.83 (0.73–0.93)	151	0.91 (0.77–1.07)	114	0.73 (0.6–0.88)
Craniosynostosis (Q75.0)	3891	12.1 (11.7–12.5)	2335	14.1 (13.6–14.7)	1556	10 (9.5–10.5)
Jeune syndrome (Q77.2)	8	0.02 (0.01–0.05)	7	0.04 (0.02–0.09)	1	0.01 (0–0.04)
Achondroplasia/Hypochondroplasia (Q77.4)	163	0.51 (0.43–0.59)	88	0.53 (0.43–0.66)	75	0.48 (0.38–0.6)
Omphalocele (Q79.2)	1345	4.2 (4–4.4)	833	5 (4.7–5.4)	512	3.3 (3–3.6)
Gastroschisis (Q79.3)	77	0.24 (0.19–0.3)	31	0.19 (0.13–0.27)	46	0.3 (0.22–0.39)
Chromosomal abnormalities						
Trisomy 13 (Q91.4–91.7)	16	0.05 (0.03–0.08)	6	0.04 (0.01–0.08)	10	0.06 (0.03–0.12)
Trisomy 18 (Q91.0–91.3)	67	0.21 (0.16–0.27)	23	0.14 (0.09–0.21)	44	0.28 (0.21–0.38)
Down’s Syndrome (Q90.0–90.9)	1301	4.1 (3.8–4.3)	734	4.4 (4.1–4.8)	567	3.6 (3.3–4)
Turner’s syndrome (Q96.0–96.9)	107	0.33 (0.27–0.4)	24	0.15 (0.09–0.22)	83	0.53 (0.42–0.66)
Klinefelter’s syndrome (Q98.0–98.4)	110	0.34 (0.28–0.41)	109	0.66 (0.54–0.8)	1	0.01 (0–0.04)
Wolf-Hirschhorn syndrome (Q93.3)	12	0.04 (0.02–0.07)	6	0.04 (0.01–0.08)	6	0.04 (0.01–0.08)
Cri-du-chat syndrome (Q93.4)	52	0.16 (0.12–0.21)	22	0.13 (0.08–0.2)	30	0.19 (0.13–0.27)

**Table 2:** Ranking of selected birth defects based on increasing trends during the study period (2008–2014).

	Birth Defects	Prevalence Per 10,000							Prevalence Rate Ratio	*p*-Value
		2008	2009	2010	2011	2012	2013	2014		
	Major 69 birth defects	336.4	372.9	401.2	445.6	474.2	539.8	563.6	1.091 (1.088–1.094)	<0.001
1	Renal dysplasia	0.43	0.49	0.96	1.55	1.75	1.97	2.00	1.275 (1.211–1.343)	<0.001
2	Omphalocele	1.46	2.07	2.11	5.60	5.82	6.00	6.38	1.265 (1.23–1.302)	<0.001
3	Congenital hip dislocation	41.79	44.96	48.03	58.23	66.27	95.86	103.67	1.185 (1.177–1.194)	<0.001
4	Reduction deformity, lower limbs	0.94	1.24	1.02	1.66	1.65	3.85	1.61	1.184 (1.133–1.237)	<0.001
5	Atrial septal defect	83.20	103.68	116.36	141.49	157.82	179.58	188.85	1.143 (1.138–1.149)	<0.001
6	Craniosynostosis	7.34	9.82	10.15	12.77	16.30	16.13	12.38	1.108 (1.09–1.126)	<0.001
7	Spina bifida	6.03	6.41	7.51	9.55	10.71	9.00	10.98	1.103 (1.082–1.124)	<0.001
8	Renal agenesis	2.00	2.34	2.59	3.63	2.97	3.16	3.97	1.102 (1.067–1.138)	<0.001
9	Undescended testis	45.56	51.80	56.61	58.32	66.88	72.05	74.32	1.082 (1.072–1.093)	<0.001
10	Single ventricle	0.82	1.06	1.11	1.29	1.59	1.28	1.26	1.072 (1.019–1.128)	0.007
